# A photoelectrochemical aptasensor based on double Z-scheme α-Fe_2_O_3_/MoS_2_/Bi_2_S_3_ ternary heterojunction for sensitive detection of circulating tumor cells

**DOI:** 10.3389/fbioe.2024.1372688

**Published:** 2024-03-07

**Authors:** Kai Feng, Ya Di, Meng Han, Weitao Yan, Yulin Guo, Xiangqian Huai, Yimin Wang

**Affiliations:** ^1^ The First Hospital of Qinhuangdao, Qinhuangdao, China; ^2^ College of Food Science and Technology, Hebei Agricultural University, Baoding, China

**Keywords:** photoelectrochemical aptasensor, dual Z-scheme, α-Fe_2_O_3_/MoS_2_/Bi_2_S_3_ ternary heterojunction, aptamer, circulating tumor cells

## Abstract

A novel photoelectrochemical (PEC) aptasensor based on a dual Z-scheme α-Fe_2_O_3_/MoS_2_/Bi_2_S_3_ ternary heterojunction for the ultrasensitive detection of circulating tumor cells (CTCs) was developed. The α-Fe_2_O_3_/MoS_2_/Bi_2_S_3_ nanocomposite was prepared via a step-by-step route, and the photoproduced electron/hole transfer path was speculated by conducting trapping experiments of reactive species. α-Fe_2_O_3_/MoS_2_/Bi_2_S_3_-modified electrodes exhibited greatly enhanced photocurrent under visible light due to the double Z-scheme charge transfer process, which met the requirement of the PEC sensor for detecting larger targets. After the aptamer was conjugated on the photoelectrode through chitosan (CS) and glutaraldehyde (GA), when MCF-7 cells were presented and captured, the photocurrent of the PEC biosensing system decreased due to steric hindrance. The current intensity had a linear relationship with the logarithm of MCF-7 cell concentration ranging from 10 to 1×10^5^ cells mL^−1^, with a low detection limit of 3 cell mL^−1^ (S/N = 3). The dual Z-scheme α-Fe_2_O_3_/MoS_2_/Bi_2_S_3_ ternary heterojunction-modified PEC aptasensor exhibited high sensitivity and excellent specificity and stability. Additionally, MCF-7 cells in human serum were determined by this PEC aptasensor, exhibiting great potential as a promising tool for clinical detection.

## 1 Introduction

Circulating tumor cells (CTCs) are a dependable biomarker for cancer diagnosis, detection, and prediction. They are released from primary or metastatic sites of tumors and circulate through peripheral blood to distant body regions (Hong and Zu, 2013; Tang et al., 2016; Wang et al., 2023a; Gong et al., 2023). Quick, inexpensive, and highly sensitive techniques to identify CTCs are urgently needed. Various conventional strategies have been established for the detection of CTCs including the immunomagnetic bead approach (Den Toonder, 2011), reverse transcriptase polymerase chain reaction (RT-PCR)-based technique (Dirix et al., 2009), enzyme-linked immunosorbent immunoassay (ELISA) (Van der Auwera et al., 2010), and fluorescence spectroscopy (Yang et al., 2018). Nevertheless, complicated operations, expensive instruments, and low sensitivity limit these methods for CTC-based clinical diagnostics. The photoelectrochemical (PEC) aptasensor, as a novel and quickly evolving technique, has found widespread use in trace analyses as an efficient method. The PEC aptasensor delivers reasonable specificity between the aptamers and target analytes (Zhong et al., 2023). As “chemical antibodies,” aptamers are single-stranded oligonucleotide sequences synthesized from the SELEX process (Stoltenburg et al., 2007; Fang and Tan, 2010; Yi et al., 2023; Zhao et al., 2023). Meanwhile, the PEC aptasensor has a high sensitivity because the excitation light source and current signal are separated completely, which minimizes interference between the input and output signals (Osterloh, 2013). Additionally, the PEC aptasensor offers exceptional benefits including simplicity, low cost, and easy integration by integrating a relatively simple optical and electrochemical instrument (Freeman et al., 2013; Yue et al., 2013). However, in order to achieve the very sensitive detection of CTCs, PEC aptasensors require a strong photocurrent because of the dielectric and relatively large size of CTCs.

Currently, semiconductors are preferred as photoactive materials for the PEC aptasensor because of their exceptional photocurrent enhancement. Molybdenum disulfide (MoS_2_) is a typical photoactive material, which has a band gap is approximately 1.8 eV (Wu et al., 2017) and energy levels that match the visible region of the solar spectrum, making it efficient for visible-light harvesting (Li et al., 2011; Hong et al., 2014). However, the challenges related to undesired photo-generated carrier (electron/hole, e−/h+) lifetimes may limit its PEC performance (Pei et al., 2019). Constructing a heterostructure (or heterojunction) with other semiconductors is considered the most efficient strategy (Liu et al., 2017; Han et al., 2018). To further boost the light utilization and electron–hole pair separation, Z-scheme heterojunction has been carried out using multiple semiconductors with well-matched band structures, which exhibits a distinct photocatalytic redox ability (Saravanakumar and Park, 2021; Yu et al., 2021). Bismuth trisulfide (Bi_2_S_3_), with a direct band gap (1.3–1.7 eV), is also ideally suited to absorb visible light and particularly well-matched with MoS_2_ nanosheets for the construction of Z-scheme heterojunction in PEC analysis. For example, Q.A. Drmosh prepared Z-scheme Bi_2_S_3_/MoS_2_/TiO_2_ nanotube-based photoelectrodes with enhanced visible light absorption and increased charge lifetime (Wang Q. et al., 2023). Similarly, hematite (α-Fe_2_O_3_), with its band gap (1.9–2.2 eV), nontoxic nature, and excellent and chemical stability, is also a promising photocatalyst in the visible-light region (Zhang Z. et al., 2020; Wheeler et al., 2012). In photocatalysis, the combination of α-Fe_2_O_3_ and MoS_2_ (α-Fe_2_O_3_/MoS_2_) is also a suitable candidate to use as a Z-scheme heterojunction. Guo and Xing designed a hollow flower-like polyhedral α-Fe_2_O_3_/MoS_2_/Ag Z-scheme heterojunction that demonstrated excellent photocatalytic degradation for 2,4-DCP (Guo et al., 2020). To enhance the activity of Z-scheme photocatalysts even more, the double Z-scheme photocatalytic system coupling of three or more semiconductors has gained extensive attention in photocatalysts, which enhanced visible light absorption and achieved more efficient charge carrier separation and transfer (Jiang et al., 2018).

Herein, we presented a novel PEC aptasensor based on a dual Z-scheme α-Fe_2_O_3_/MoS_2_/Bi_2_S_3_ ternary heterojunction for the ultrasensitive detection of CTCs. The α-Fe_2_O_3_/MoS_2_/Bi_2_S_3_ nanocomposite was prepared via a step-by-step route, and α-Fe_2_O_3_/MoS_2_/Bi_2_S_3_-modified electrodes exhibited greatly enhanced photocurrent under visible light. The photoproduced electron/hole transfer path was speculated by conducting trapping experiments of reactive species to demonstrate the charge transfer process. After the aptamer was conjugated on the photoelectrode, MCF-7 cells were captured through a specific immunoreaction between the aptamer and tumor, leading to the decrease in photocurrent due to steric hindrance. The evolution of the current signal could be reflected directly through the concentration of MCF-7 cells. The fabricated PEC aptasensor showed excellent sensitivity, stability, and selectivity. Additionally, MCF-7 cells in human serum were determined by this PEC aptasensor, which exhibited great potential in clinical detection.

## 2 Materials and methods

### 2.1 Materials and apparatus

Ferrous sulfate hydrate (FeSO_4_·7H_2_O), urea, ethanol, ammonium molybdate tetrahydrate ((NH_4_)_6_MoO_24_·4H_2_O), thiourea, bismuth nitrate pentahydrate (Bi(NO_3_)_3_·5H_2_O), glacial acetic acid, glutaraldehyde (50%, GA), ascorbic acid (AA), isopropanol (IPA), p-benzoquinone (BQ), methylene blue (MB), and chitosan (CS) were purchased from Aladdin Reagent Company (Shanghai, China). Fluorine-doped tin oxide (FTO) glass was obtained from South China Xiangcheng Technology Co., Ltd. Oligonucleotides and bovine serum albumin (BSA) were purchased from Sangon Biotech Co., Ltd. (Shanghai, China), and all chemical reagents were analytical grade without further purification.

Aptamer DNA (Apt-DNA):

$${\text{NH}}_{2}-C_{12}-\text{CACTACAGAGGTTGCGTCTGTCCCACGTTGTCA}\text{TGGG GGGTTGGCCTG}$$

All the electrochemical measurements were carried out on a CHI 760E electrochemical workstation (Shanghai Chenhua Instrument Co., Ltd., China) with a three-electrode system composed of FTO as the working electrode, a platinum electrode as the counter electrode, and a saturate Ag/AgCl electrode as the reference electrode. Electrochemical impedance spectroscopy (EIS) and cyclic voltammetry (CV) were performed in 5 mM K_3_Fe(CN)_6_/K_4_Fe(CN)_6_ (0.1 M KCl) as the supporting electrolyte.

### 2.2 Preparation of the double Z-scheme α-Fe_2_O_3_/MoS_2_/Bi_2_S_3_ ternary heterojunction

Flower-like α-Fe_2_O_3_ with nanorod petals was prepared as depicted in a previous report with minor modification (Wang et al., 2023c). First, 2.28 g of FeSO_4_·7H_2_O and 0.6 g of urea were dissolved in 100 mL mixed solution (V_H2O_:V_C2H5OH_ = 4:1) and sonicated for 10 min. Then, the mixed solution was transferred into a 250-mL three-necked flask to reflux at 90°C for 6 h. After precipitation and drying at 60°C for 24 h, the reddish brown FeOOH powder was prepared. Subsequently, the α-Fe_2_O_3_ nanorods were obtained by the calcination of the prepared FeOOH at 500°C for 3 h in a Laboratory Muffle stove.

α-Fe_2_O_3_/MoS_2_ nanocomposites were successfully prepared via a hydrothermal route. First, 0.1234 g of (NH_4_)_6_MoO_24_·4H_2_O and 0.2284 g of thiourea were dissolved in 35 mL of distilled water and stirred for 30 min. Then, 0.357 g of α-Fe_2_O_3_ was added to the above solution under stirring for 30 min. Subsequently, the obtained solution was transferred to a 50-mL Teflon-sealed autoclave and heated to 200°C for 6 h. After being cooled to room temperature, the α-Fe_2_O_3_/MoS_2_ nanocomposites were obtained after being centrifuged and washed three times.

α-Fe_2_O_3_/MoS_2_/Bi_2_S_3_ nanocomposites were successfully prepared by a hydrothermal process. First, 0.0236 g of thiourea was added in 25 mL distilled water and stirred for 3 min. Then, 0.076 g of Bi(NO_3_)_3_·5H_2_O was added to the above solution and stirred for 20 min. Then, 0.04 g of α-Fe_2_O_3_/MoS_2_ nanocomposites was added and stirred at 180°C for 20 min. After being cooled to room temperature, the α-Fe_2_O_3_/MoS_2_/Bi_2_S_3_ nanocomposites were obtained after being centrifuged and washed. The product was dried in an oven at 60°C for 24 h for the next experiment.

### 2.3 Fabrication of the PEC aptasensor and PEC detection of CTCs

**SCHEME 1:**
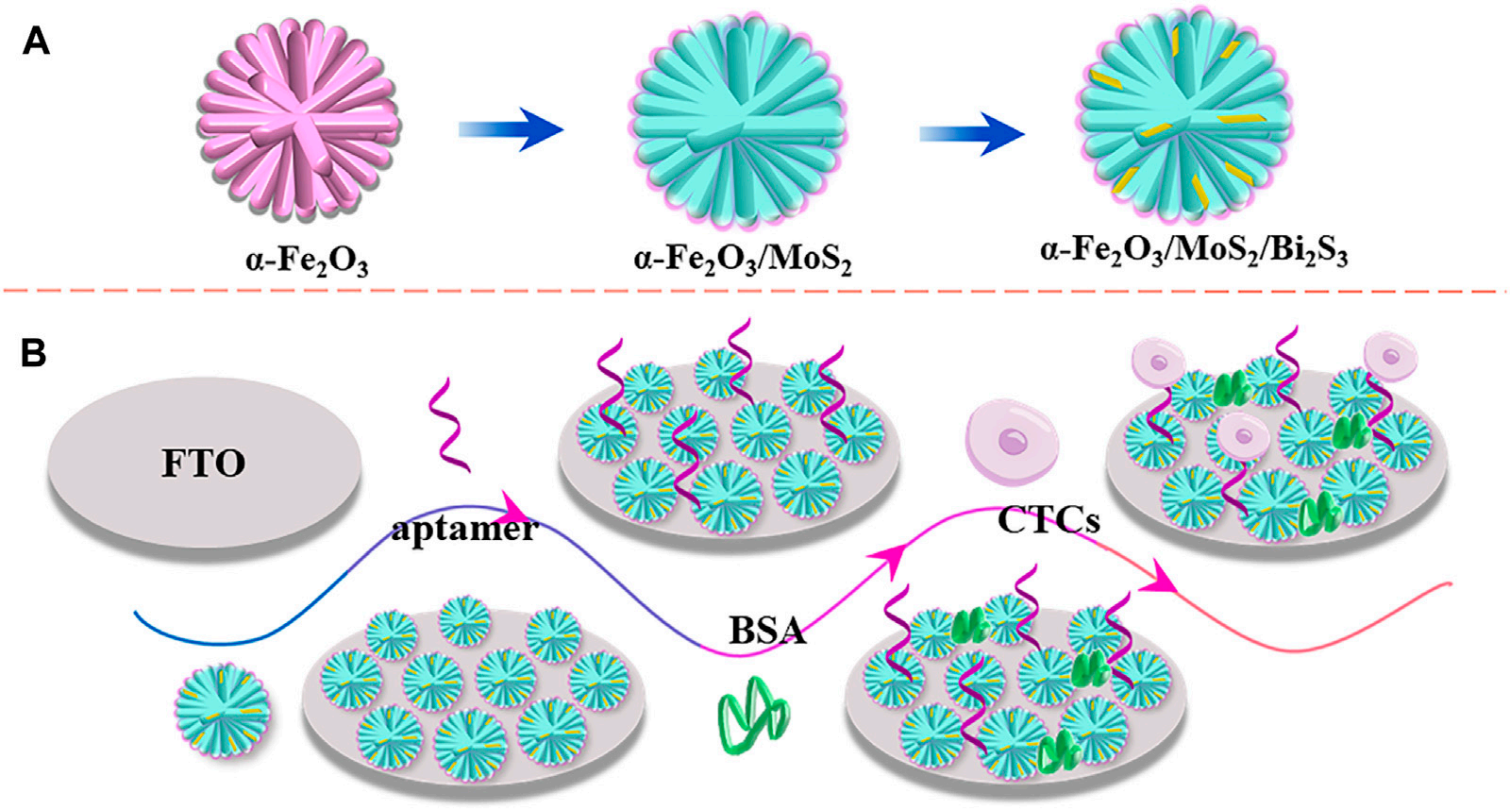
Schemata of the preparation of the α-Fe_2_O_3_/MoS_2_/Bi_2_S_3_ ternary heterojunction **(A)** and fabrication of the PEC aptasensor **(B)**.

The PEC aptasensor based on a direct dual Z-scheme α-Fe_2_O_3_/MoS_2_/Bi_2_S_3_ ternary heterojunction for the ultrasensitive detection of CTCs is shown in Scheme 1. First, 20 μL (2 mg mL^−1^) of α-Fe_2_O_3_/MoS_2_/Bi_2_S_3_ nanocomposites were dropped to the surface of FTO, and 20 μL of mixture solution containing chitosan and acetic acid (chitosan/acetic acid = 1%, w/v) was added on the electrode surface of FTO/α-Fe_2_O_3_/MoS_2_/Bi_2_S_3_. After being dried at 37°C, the FTO/α-Fe_2_O_3_/MoS_2_/Bi_2_S_3_ electrode was immersed in GA solution (0.2%) and incubated for 30 min. Then, 20 μL of aptamer DNA (5 μM) was dropped onto the electrode and incubated for 40 min at 37°C. Subsequently, 20 μL of BSA (1%) was used to block the nonspecific binding sites, and the capture electrode FTO/α-Fe_2_O_3_/MoS_2_/Bi_2_S_3_/CS/GA/BSA was constructed. A volume of 20 μL of MCF-7 cell solution with different concentrations was dropped onto the electrode surface and incubated for 120 min at 37°C. Finally, the PEC response of the biosensor was recorded in 10 mL of PBS (0.01 M, pH 7.4) containing ascorbic acid (AA, 0.14 mol L^−1^) under visible light irradiation using a LED lamp (excitation wavelength, 450 nm; 100 W) with on–off light switching of 10 s.

## 3 Results and discussion

### 3.1 Characterization of the α-Fe_2_O_3_/MoS_2_/Bi_2_S_3_ ternary heterojunction

**FIGURE 1:**
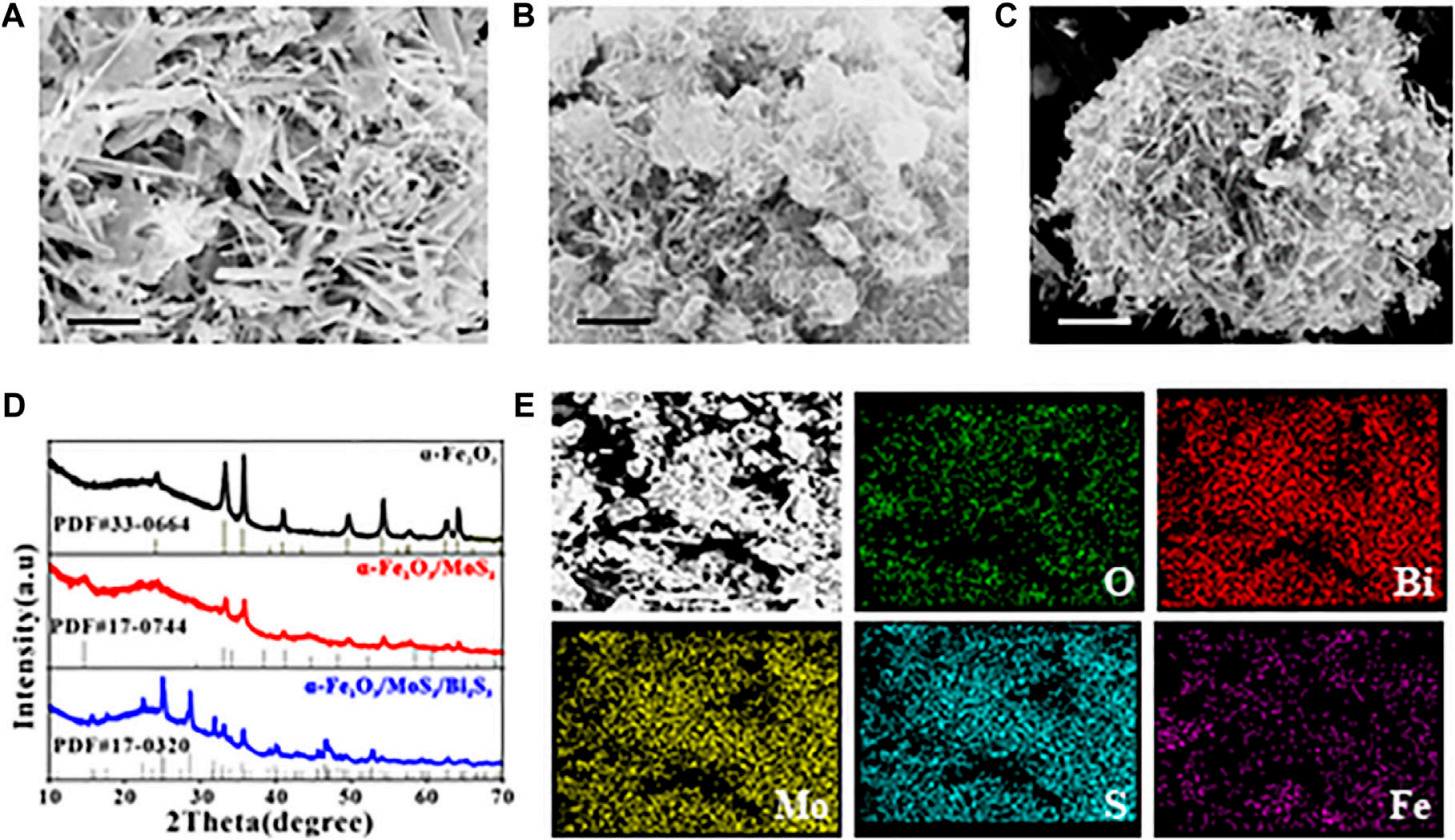
SEM images of **(A)** α-Fe_2_O_3_, **(B)** α-Fe_2_O_3_/MoS_2_, and **(C)** α-Fe_2_O_3_/MoS_2_/Bi_2_S_3_. **(D)** XRD patterns of α-Fe_2_O_3_, α-Fe_2_O_3_/MoS_2_, and α-Fe_2_O_3_/MoS_2_/Bi_2_S_3_. **(E)** SEM-EDS mapping of α-Fe_2_O_3_/MoS_2_/Bi_2_S_3_. Scale bar for **(A–C)** is 1 μm.

SEM was used to analyze the morphology of the as-synthesized samples. As shown in Figure 1A, the SEM image of α-Fe_2_O_3_ displayed a uniform flower-like nanostructure, and the nanorod petal was approximately 3–4 µm in length. The SEM image of α-Fe_2_O_3_/MoS_2_ (Figure 1B) revealed that α-Fe_2_O_3_ was encapsulated in MoS_2_ nanosheets and exhibited ripples, which indicated the formation of the α-Fe_2_O_3_/MoS_2_ heterojunction. Subsequently, Bi_2_S_3_ grew in the layered MoS_2_, and the α-Fe_2_O_3_/MoS_2_/Bi_2_S_3_ ternary heterojunction exhibited an icicle flower-like structure, as shown in Figure 1C. X-ray diffraction (XRD) patterns were used to characterize the α-Fe_2_O_3_/MoS_2_/Bi_2_S_3_ ternary heterojunction. Figure 1D shows the characteristic diffraction peaks that correspond to the JCPDS card No. 33–0664 α-Fe_2_O_3_, respectively. Additionally, three peak representatives (14.13°, 28.47°, and 32.91°) which belonged to the (002), (004), and (100) crystal planes of MoS_2_ (JCPDS card No. 75–1539), respectively, proved the formation of MoS_2_. Meanwhile, a few prominent peaks of Bi_2_S_3_ appeared based on JCPDS card No. 17–0320. These illustrated the formation of the α-Fe_2_O_3_/MoS_2_/Bi_2_S_3_ ternary heterojunction. As expected, the element mapping images (Figure 1E) showed the distribution of O, Bi, Mo, S, and Fe, offering direct evidence of the effective achievement of α-Fe_2_O_3_/MoS_2_/Bi_2_S_3_ ternary heterojunction. The UV-vis absorption spectra of α-Fe_2_O_3_, α-Fe_2_O_3_/Bi_2_S_3_, and α-Fe_2_O_3_/MoS_2_/Bi_2_S_3_ were investigated as described in Supplementary Figure S1. Both MoS_2_ and Bi_2_S_3_ presented a broad absorption spectrum across the visible light region. For the α-Fe_2_O_3_/MoS_2_/Bi_2_S_3_ heterojunction, α-Fe_2_O_3_ also enhanced its absorption ability in visible light, which would lead to an increase in photocatalytic activity.

### 3.2 Photocatalytic mechanism of the α-Fe_2_O_3_/MoS_2_/Bi_2_S_3_ ternary heterojunction

The band gap energy (Eg) of α-Fe_2_O_3_ (2.1 eV), MoS_2_ (1.38 eV), and Bi_2_S_3_ (1.41 eV) was investigated by UV–vis diffuse reflectance spectroscopy, and the flat-band potentials (α-Fe_2_O_3_, 0.69 eV; MoS_2_, −0.4 eV; Bi_2_S, 0.59 eV; all vs. SSCE) were derived using Mott–Schottky plots, as shown in Supplementary Figure S2. Their valence bands (VBs) were 0.93 eV, −0.16 eV, and −0.35 eV, respectively, which was obtained based on the following formula: VB = CB + Eg. The trapping experiments of reactive species in this photocatalytic process were carried out. In this work, IPA (radical •OH scavenger) and BQ (radical •O^2−^ scavenger) were employed as quenchers in the degradation experiment of methylene blue (MB), as shown in Supplementary Figure S3. During this photocatalytic process, both BQ and IPA significantly reduced the degradation rate of MB, demonstrating that a larger amount of •OH and •O^2−^ on the surface of α-Fe_2_O_3_/MoS_2_/Bi_2_S_3_ was involved in the degradation of MB. The standard potential of the OH^−^/•OH pair (+2.40 eV vs. NHE) was lower than the VB position of α-Fe_2_O_3_ and higher than the VB position of both MoS_2_ and Bi_2_S_3_. We could speculate that only h+ of α-Fe_2_O_3_ reacted with OH− or H_2_O to form •OH. Meanwhile, the standard potential of the O_2_/•O^2−^pair (−0.33 eV vs. NHE) was more positive than that of Bi_2_S_3_ and more negative than the CB of both α-Fe_2_O_3_ and MoS_2_. It was concluded that •O^2−^was more possible to be produced by Bi_2_S_3_.

**FIGURE 2:**
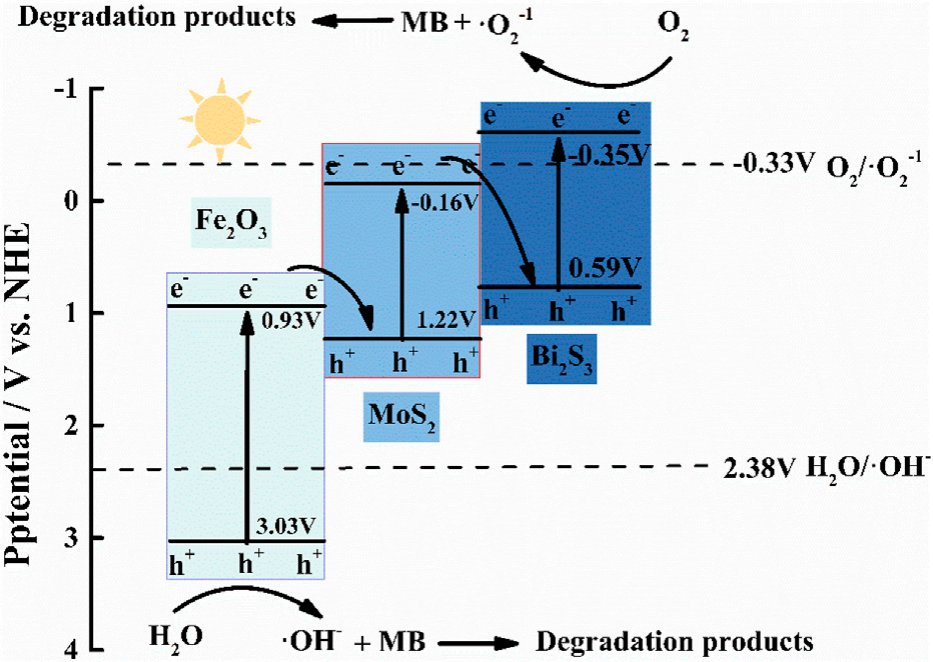
Proposed charge transfer and photocatalytic mechanisms of the double Z-scheme α-Fe_2_O_3_/MoS_2_/Bi_2_S_3_ ternary heterojunction.

Based on these, the transfer pathway of electrons in α-Fe_2_O_3_/MoS_2_/Bi_2_S_3_ is shown in Figure 2. Under visible irradiation, photo-generated e−/h+ was produced on the CB and VB of α-Fe_2_O_3_, Bi_2_S_3_, and MoS_2_. The e− in the CB of α-Fe_2_O_3_ and MoS_2_ transferred to the VB of MoS_2_ and Bi_2_S_3_ to recombine with the h+, respectively. This resulted in the accumulation of high-energy e− and h + on the VB of Bi_2_S_3_ and the CB of α-Fe_2_O_3_, where they participated in photocurrent production. This double Z-scheme heterojunction promoted the detecting photocurrent intensity in the PEC aptasensor because it effectively inhibited the recombination of electron–hole pairs and absorbed sufficient light.

### 3.3 Characterization of the PEC aptasensor

**FIGURE 3:**
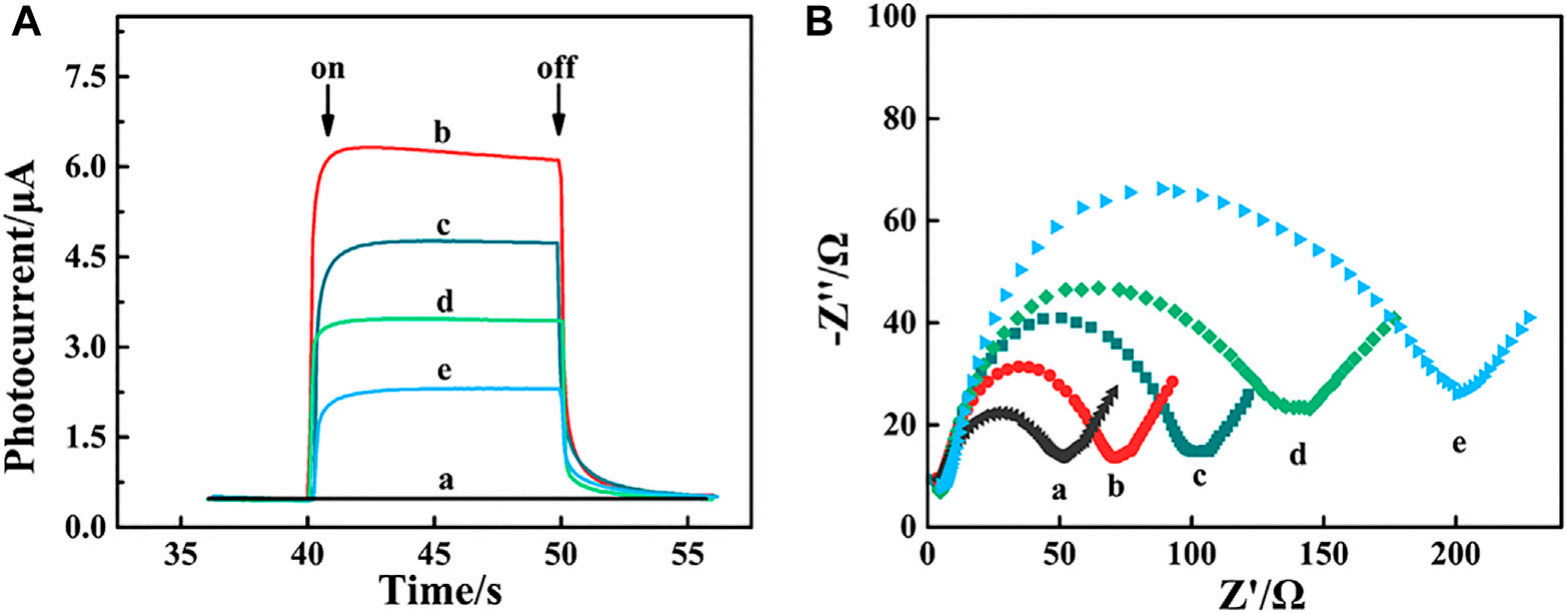
Photocurrent response **(A)** and EIS spectrum **(B)** of FTO electrode. (a), α-Fe_2_O_2_/MoS_2_/Bi_2_S_2_ heterojunction (b), α-Fe_2_O_2_/MoS_2_/Bi_2_S_2_ heterojunction/Apt-DNA (c) and α-Fe_2_O_3_/MoS_2_/Bi_2_S_3_ /Apt-DNA/BSA (d) and α-Fe_2_O_2_/MoS_2_/Bi_2_S_2_/Apt-DNA/BSA/MCF-7 cell.

As shown in Figure 3A, the photocurrent of FTO/α-Fe_2_O_3_/MoS_2_/Bi_2_S_3_ (curve b) was much larger than that of the naked FTO electrode (curve a) due to the creation of ternary heterojunctions with high light absorption and photoelectric conversion efficiency. Steric hindrance caused a reduction in photocurrents after Apt-DNA (curve c), BSA (curve d), and MCF-7 cell (curve e) were assembled on the photoelectrode of FTO/α-Fe_2_O_3_/MoS_2_/Bi_2_S_3_. These demonstrated that the PEC aptasensor was successfully constructed. Meanwhile, electrochemical impedance spectroscopy (EIS) was also carried out to validate this process. The diameter of the high-frequency semicircle in the Nyquist plot corresponded to the electron transfer resistance (R_et_) of the electrode surface (Luo et al., 2022). As shown in Figure 3B, the R_et_ value of the naked FTO electrode was small (curve a), and it drastically decreased (curve b) when the α-Fe_2_O_3_/MoS_2_/Bi_2_S_3_ heterojunction was dropped on the FTO electrode. Subsequently, when the Apt-DNA (curve c), BSA (curve d), and MCF-7 cell (curve e) were continuously assembled on the FTO/α-Fe_2_O_3_/MoS_2_/Bi_2_S_3_ surface, they led to an increase in R_et_ because they impeded the diffusion of electrons to the electrode surface, indicating their successful immobilization.

### 3.4 Optimization of PEC measurement conditions

**FIGURE 4:**
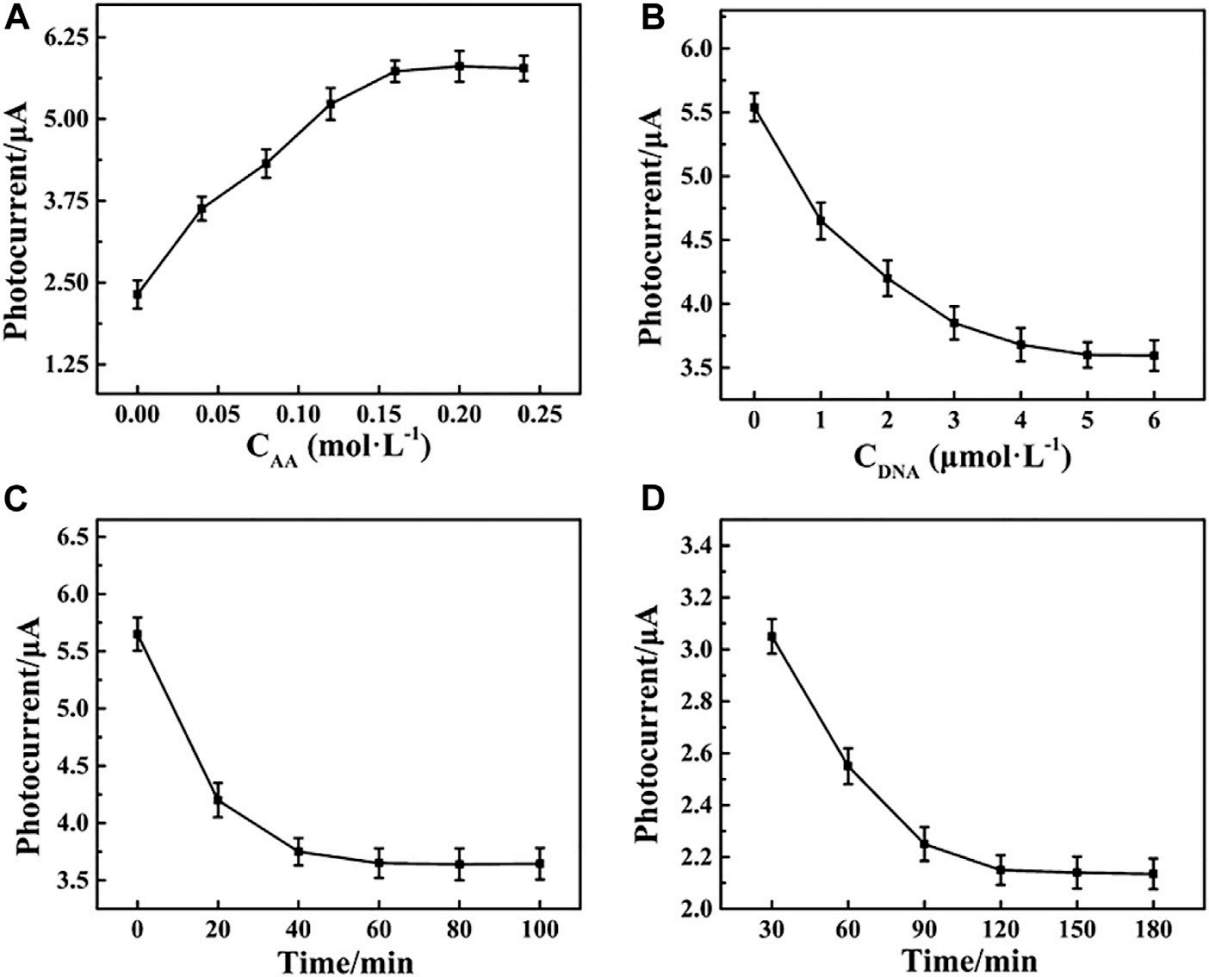
Effects of the **(A)** concentration of Apt-DNA, **(B)** amount of AA, **(C)** incubation time of the Apt-DNA with the photoelectrode, and **(D)** captured electrode with MCF-7 cells.

A number of parameters, including the concentration of Apt-DNA, the amount of AA, and the incubation time of the Apt-DNA with the photoelectrode and captured electrode with MCF-7 cells, were optimized. As shown in Figure 4A, the photocurrent response peaked at 0.15 mol L^−1^, and no obvious change was observed at higher concentrations. As a result, the concentration of AA in all subsequent experiments was 0.15 mol L^−1^. The impact of Apt-DNA concentration on the PEC response of the biosensor is shown in Figure 4B. The photocurrent decreased as the concentration of Apt-DNA increased up to 5 M, after which there was no obvious change, indicating Apt-DNA saturation. Accordingly, 5 µM of Apt-DNA was used in all subsequent experiments. The immobilization time is also shown in Figure 4C. The photocurrent decreased in the range from 0 to 60 min and then remained constant. It was that the amount of Apt-DNA was saturated after a certain time. Meanwhile, the incubation time of Apt-DNA with the captured electrode was also examined, as shown in Figure 4D. The ideal duration was found to be approximately 120 min. Under optimal conditions, the photocurrent was large and stable, which would be performed for subsequent experiments.

### 3.5 Detection performance of the PEC aptasensor

**FIGURE 5:**
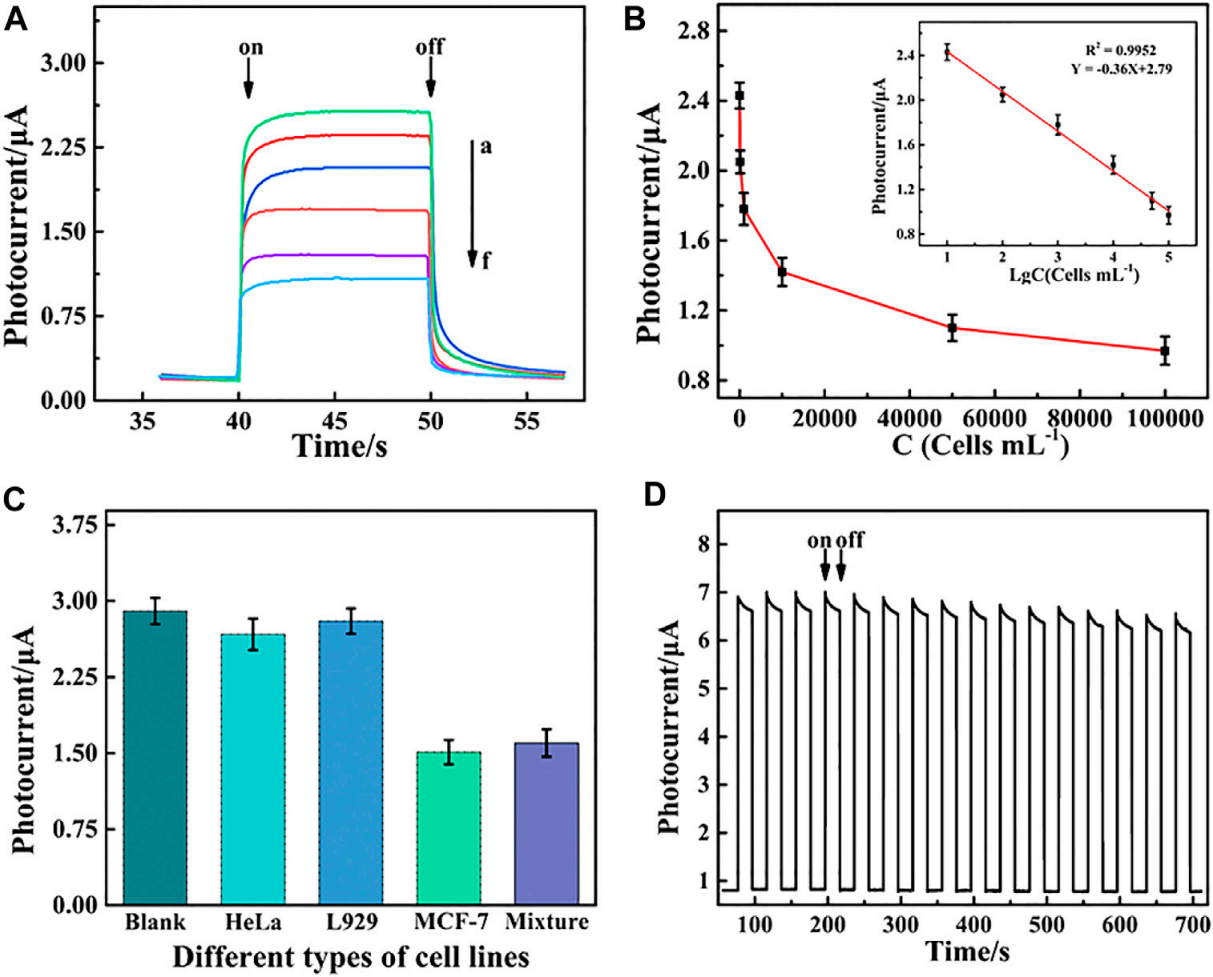
**(A)** Photocurrent responses of the PEC aptasensor toward MCF-7 cells and **(B)** relationship of the PEC signal and cell concentration at different concentrations ranging from 10 to 1×10^5^ cells mL^−1^. Inset of **(B)** shows linear relationship between the change in photocurrent intensity (ΔI) and the logarithm value of the MCF-7 cell concentration. **(C)** Selectivity of PEC detection for MCF-7 cells including the blank, HeLa, L929, MCF-7 cells, and mixture cells containing HeLa, L929, and MCF-7. **(D)** Stability of the PEC biosensor under repeated light irradiation from 0 to 700 s.

The PEC response decreased with an increase in MCF-7 cell concentrations due to steric hindrance (Figure 5A). The decrease in photocurrent intensity demonstrated a good linear relationship with the logarithm of the MCF-7 cell concentration in the range from 10 to 1×10^5^ cells mL^−1^ (Figure 5B). The linear regression equation was y = −0.36 lgC cells+2.79 (C cells, cell mL^−1^) with a correlation coefficient (*R* ^2^) of 0.9952 (n = 3) and a low detection limit of 3 cell mL^−1^ (S/N = 3). Therefore, the PEC aptasensor exhibited an ultrasensitive detection of MCF-7 cells compared with the other biosensors given in Table 1.

**TABLE 1:** Comparison of the performance of the PEC aptasensor with other methods for CTC detection.

Method	Linear range	Detection limit	Reference
Electrochemistry	18–1.5 × 10^6^ cells/ mL^−1^	6 cells/ mL^−1^	Zhang et al. (2020b)
PEC	10^2^–5 × 10^5^ cells/ mL^−1^	15 cells/ mL^−1^	Ding et al. (2023)
Fluorescence	10–10^5^ cells/ mL^−1^	3 cells/ mL^−1^	Chen et al. (2021)
Chemiluminescence	10^2^–1 × 10^6^ cells/ mL^−1^	15 cells/ mL^−1^	He et al. (2015)
Colorimetry	10^2^–10^5^ cells/ mL^−1^	12 cells/ mL^−1^	Wang et al. (2018)
This work	10–10^5^ cells/ mL^−1^	3 cells/ mL^−1^	

### 3.6 Application of the PEC aptasensor in real samples

To assess the application potential, the prepared PEC aptasensor was used to detect MCF-7 cells in real samples. MCF-7 cells with different concentrations (10, 50, 100, 500, and 1,000 cells mL^−1^) were spiked into serum samples for the assay. The recoveries of MCF-7 were between 92% and 107.6% with a relative standard deviation (RSD) from 5.7% to 7.8% (Table 2), demonstrating great potential for the detection of CTCs in real samples.

**TABLE 2:** Spiked detection of CTCs in serum samples (n = 6).

Add (cells mL^−1^)	Detected (cells mL^−1^)	Recovery (%)	RSD (%)
50	46	92	6.2
100	93	93	7.8
500	538	107.6	6.5
1,000	1,053	105.3	5.7

## 4 Conclusion

In summary, we developed a dual Z-scheme PEC aptasensor based on the α-Fe_2_O_3_/MoS_2_/Bi_2_S_3_ ternary heterojunction for the ultrasensitive detection of CTCs. The α-Fe_2_O_3_/MoS_2_/Bi_2_S_3_ ternary nanocomposite was prepared via a step-by-step route, and the analysis of radical trapping experiments confirmed that the active species •O^2−^, h^+^, and •OH were produced in the α-Fe_2_O_3_/MoS_2_/Bi_2_S_3_ photocatalytic system. The mechanism analysis demonstrated that the charge transfer of the α-Fe_2_O_3_/MoS_2_/Bi_2_S_3_ nanocomposite followed a dual Z-scheme route, which exhibited a significant enhanced photocurrent under visible light, resulting in improved visible light absorption, increased surface area, and enhanced separation efficiency of photo-generated electron–hole pairs. The constructed PEC aptasensor offered a linear PEC response, with the CTC concentration ranging from 10 to 1×10^5^ cells mL^−1^ and a low detection limit of 3 cell mL^−1^ (S/N = 3). Additionally, MCF-7 cells in human serum were determined by this PEC aptasensor, which exhibited great potential in clinical detection.

## Data Availability

The original contributions presented in the study are included in the article/Supplementary Material; further inquiries can be directed to the corresponding authors.
