# Supplementary material for: A photoelectrochemical aptasensor based on double Z-scheme α-Fe2O3/MoS2/Bi2S3 ternary heterojunction for sensitive detection of circulating tumor cells
Source: Front Bioeng Biotechnol. 2024 Mar 7;12:1372688. doi: 10.3389/fbioe.2024.1372688 (PMC10956413; doi:10.3389/fbioe.2024.1372688)
Supplement: Supplementary file 1 [file DataSheet1.PDF]

Supplementary Material

1 Supplementary Figures

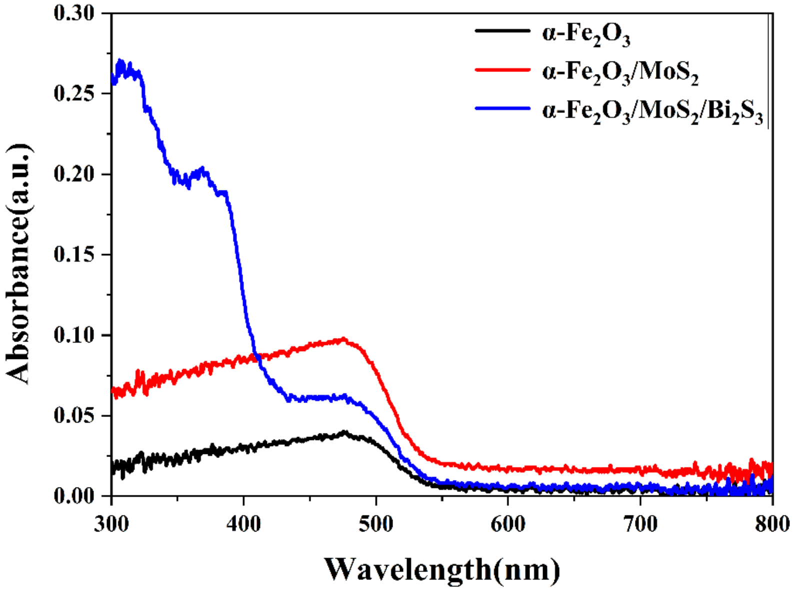

Fig S1. UV–vis DRS spectra of α-Fe2O3, α-Fe2O3/MoS2 and α-Fe2O3/MoS2/Bi2S3

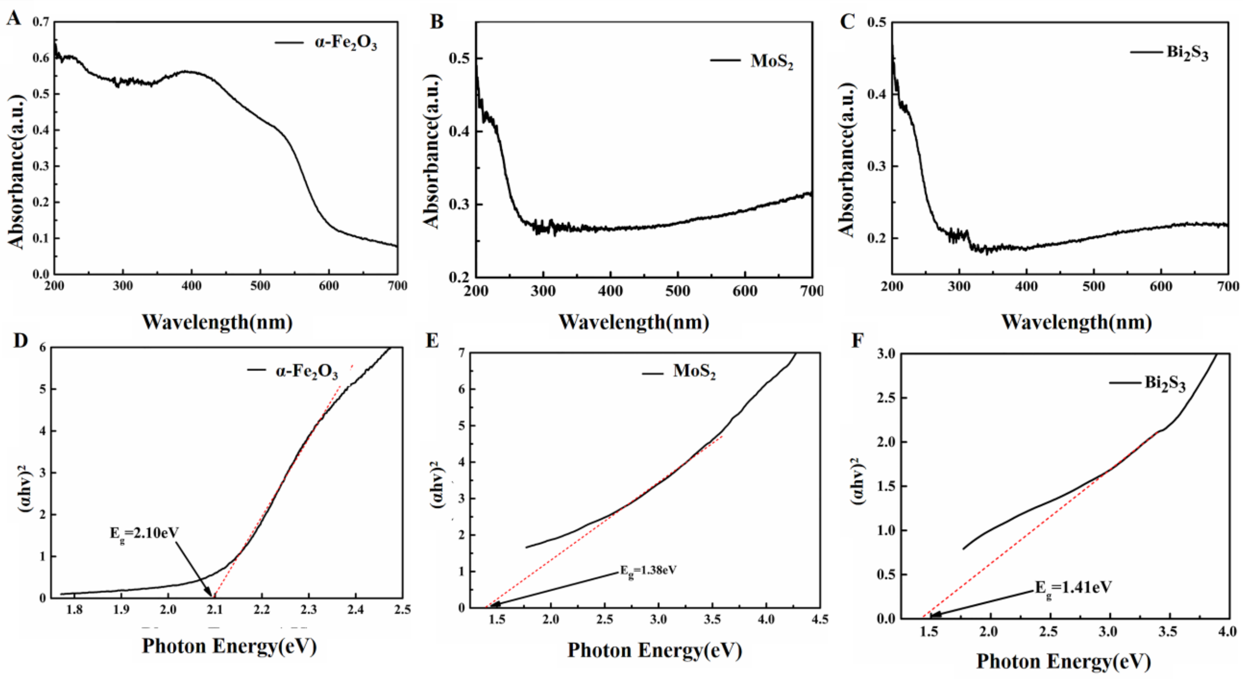

Fig S2. DRS spectra of (A)α-Fe2O3, (B) MoS2, (C) Bi2S3 and corresponding Tauc' plots of (D)α-Fe2O3, (E) MoS2, (F) Bi2S3.

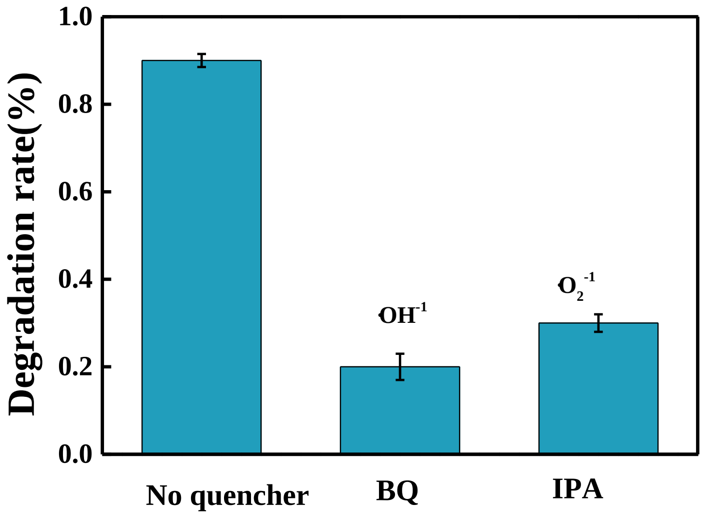

Fig S3 IPA and BQ, radical •O2− as quenchers of effect on degradation of MB.
